# Goal-directed therapy: what we know and what we need to know

**DOI:** 10.1186/s13741-015-0012-1

**Published:** 2015-02-05

**Authors:** Jason B O’Neal, Andrew D Shaw

**Affiliations:** Department of Anesthesia, Critical Care and Pain Medicine, Beth Israel Deaconess Medical Center, Harvard Medical School, Boston, MA USA; Department of Anesthesiology, Vanderbilt University Medical Center, Nashville, TN USA

## Abstract

Goal-directed therapy (GDT) utilizes monitoring techniques to help guide clinicians with administering fluids, vasopressors, inotropes, or other treatments to patients in various clinical settings. Multiple studies have investigated the potential benefits of GDT, but no consensus on the use of GDT exists. Future trials which address fluid and inotrope choice as well as expanding the results to evaluate patient-centered outcomes in addition to survival are warranted.

## Background

Achieving hemodynamic stability during and after surgery to ensure adequate perfusion and oxygenation is a goal of every perioperative physician. We have several types of invasive and non-invasive monitoring techniques that influence our decisions to give intravenous fluids, start vasopressors, or initiate inotropes on a patient. Goal-directed therapy (GDT) utilizes these monitors to assess cardiovascular performance and thus guides the clinician to intervene as necessary based on a predetermined algorithm. Many investigators have conducted studies to examine the potential benefits of GDT in surgical patients and also in the intensive care unit (ICU) [1,2]. Two recent large multi-center, randomized controlled trials, the Australasian Resuscitation In Sepsis Evaluation (ARISE) and Protocolized Care for Early Septic Shock (ProCESS) studies, examined GDT in early septic shock [3,4]. The ARISE study found no reduction in all-cause mortality at 90 days, and the ProCESS study showed no improvement in outcomes including 60-day in-hospital mortality, 90-day mortality, 1-year mortality, or the need for organ support. A paper in *Critical Care Medicine*, using a simulation model of a tertiary care hospital in the United Kingdom, found a cost benefit of goal-directed therapy. The short-term model suggested that GDT reduced the hospital length of stay and was also associated with fewer complications [5]. Although there is a vast number of publications on GDT, and a possible cost benefit, no general consensus on the use of GDT exists. A study published in *JAMA*, the Optimisation of Cardiovascular Management to Improve Surgical Outcome (OPTIMISE) trial, examined the effect of GDT in high-risk gastrointestinal (GI) surgical patients on outcomes following surgery [6].

The OPTIMISE trial was a multi-center, randomized, observer blinded trial of 734 high risk patients undergoing major GI surgery in 17 hospitals in the United Kingdom. The aim of the study was to evaluate a GDT algorithm using intravenous fluid boluses and an inotrope (dopexamine). Cardiac output was measured with the LiDCO (hemodynamic monitor), and the intervention group received non-standardized 250 cc colloid fluid boluses and were started on a dopexamine infusion at a set rate to attain an adequate stroke volume. The management between the intervention and usual care groups was similar except more colloid was administered in the intervention group, and also the intervention group received more blood products both during and after surgery.

The primary outcome of the study was 30-day moderate or major complications and mortality. This was present in 36.6% of the intervention group as compared to 43.4% in the usual care group with a relative risk (RR) of 0.84 (95% CI 0.71–1.01; *p* = 0.07). The primary outcome as well as all secondary outcomes including morbidity on day 7, infection, critical care-free days, all-cause mortality at 30/180 days, and length of stay were not significantly different between the groups. Thus, this study did not confirm previous data suggesting that GDT has an outcome benefit.

This is the largest trial of a perioperative, cardiac output-guided therapy algorithm in patients undergoing major surgery to date, and no difference was found between the GDT group and the usual care group. A recent Cochrane review also suggested that GDT does not reduce mortality perioperatively; however, it may reduce complications and hospital length of stay [1]. Interestingly, in addition to the OPTIMISE randomized clinical trial, the group conducted a systematic review which included a meta-analysis of 38 trials. This included the same 31 studies from the Cochrane review and added the results from OPTIMISE and six other studies. The meta-analysis showed a reduction in complications in the intervention group versus the control with a RR of 0.77 (95% CI 0.71–0.83; *p* < 0.001). No significance was seen in hospital stay, 28-day mortality, and 30-day mortality (RR 0.82, 95% CI 0.67–1.01; *p* = 0.06) or mortality at the longest follow-up with 267/3,215 [8.3%] in the intervention group and 327/3,160 [10.3%] in the control (RR 0.86, 95% CI 0.74–1.00; *p* = 0.06).

Although the additional studies in the Cochrane review resulted in a statistically significant reduction of complication rates, the trials included were small, outcomes were inconsistent, and criteria were quite diverse. Some of the studies were conducted over a decade ago, and clinical management is ever-changing. The fact remains that a large, randomized trial showed no difference in mortality when GDT was implemented into clinical care. OPTIMISE showed a trend in the direction which supports GDT, but more patients were likely needed to show a difference. When including 65 patients whose care was defined as non-adherent, the treatment effect was strengthened to a RR 0.8 (95% CI 0.61–0.99; *p* = 0.04). The majority of non-adherent patients had dopexamine administered either lower or higher than the pre-defined set rate or received dopexamine for less than 6 h after surgery. The trial did not address the fact that the interventional group received more blood products than the usual care group. This could indicate that patients in the GDT group lost more blood intraoperatively and may have affected the complication rate negatively.

The OPTIMISE trial and other studies on GDT are not consistent with showing harm from this intervention; however, some concerns may arise with clinicians worried about cardiac complications in patients receiving fluid boluses and inotropic agents. A meta-analysis reviewing cardiac complications in 22 randomized controlled trials observing patients receiving GDT found a reduction in cardiovascular complications and arrhythmias, and no difference in the rate of myocardial ischemia or acute pulmonary edema [7]. Another study on GDT in elderly patients with known coronary artery disease undergoing GI surgery found no difference in adverse cardiac events such as congestive heart failure, myocardial ischemia, and cardiac. This study also found that GDT was associated with a shorter ICU stay and time to discharge and faster return of GI function [8]. These findings suggest GDT to be safe when considering the potential cardiac effects of introducing boluses of intravenous fluid and increased cardiac demand with inotropes.

Most studies on GDT focus on initial complications and mortality as primary and secondary endpoints. This data may one day direct and change the standard of clinical care on patients, but there are limited studies investigating patient-centered outcomes such as disability or cognitive deficits 1 year after surgery. Long term survival may be improved in these patients as evidenced by a follow-up study conducted 15 years after the initial randomized control trial on GDT [9]. In that study, median survival was 3 years longer in the treatment group. With the aging population and government focus on cutting health-care costs, patient-centered outcomes are becoming an important and worthwhile measure of clinical care.

## Conclusion

The question of whether or not GDT is truly beneficial still remains unanswered. Assuming there is a benefit, another question which needs to be addressed is the type of fluid to administer and if the addition of an inotrope, as well as which one, is necessary for GDT to succeed. The algorithm varies between studies making it difficult to interpret the results of meta-analyses including their data. And what is the best hemodynamic goal to direct therapy? Some studies assess cardiac output or stroke volume while others use mixed venous oxygen saturation or another parameter. No single hemodynamic goal or monitoring method has been accepted across the literature [10]. To mention, OPTIMISE 2 is in the works with plans to address fluid choice with or without an inotropic agent as well as which monitor is the best option. The outcomes may need to be broadened, and studies which include disability-free survival at 1 year as well as other patient-centered outcomes should be completed. Additional studies are warranted before conclusions on GDT can be made, and a single algorithm with which clinicians should direct care may be difficult to universalize.

## Abbreviations

GDT: Goal-directed therapy
ICU: Intensive care unit
GI: Gastrointestinal
RR: Relative risk
